# Hepatoprotective effects of flavonoids from common buckwheat hulls in type 2 diabetic rats and HepG2 cells

**DOI:** 10.1002/fsn3.2390

**Published:** 2021-07-07

**Authors:** Hai Wang, Shuyan Liu, Yang Cui, Yue Wang, Yang Guo, Xiujuan Wang, Junmei Liu, Chunhong Piao

**Affiliations:** ^1^ College of Food Science and Engineering Jilin Agricultural University Changchun China; ^2^ National Engineering Laboratory for Wheat and Corn Deep Processing Changchun China

**Keywords:** antioxidant, buckwheat hull flavonoids (BHFs), hepatoprotective potential, HepG2 cells, type 2 diabetes

## Abstract

Flavonoids from common buckwheat hulls (BHFs) show significant antioxidant and antidiabetic potential. However, their hepatoprotective property is yet to be defined. This study aims to examine the hepatoprotective effect of BHFs in type 2 diabetes mellitus (T2DM) rats and chronic high glucose‐damaged HepG2 cells. Results showed that BHF treatment significantly relieves the state of insulin resistance, thereby reducing blood glucose and improving oxidative stress in T2DM rats. It is worth mentioning that BHF treatment improved diabetes‐induced liver damage disorders, manifested as the clearance of liver fat and the decline of serum alanine aminotransferase (ALT) and aspartate aminotransferase (AST) activities. In vitro, HepG2 cells pretreated with BHFs maintained higher superoxide dismutase (SOD), glutathione peroxidase (GSH‐px), and catalase (CAT) activities than the unprotected group. In parallel, compared with the unprotected group, BHFs significantly reduced the leakage of ALT and AST in pre‐protected group dose‐dependently. These results indicated that BHFs had considerable antioxidant and hepatoprotective potential and could be promising to be used as nutraceuticals and dietary supplements to prevent and/or protect against liver disorders.

## Graphical abstract

Buckwheat hull flavonoids had a considerable ability to regulate blood glucose. Buckwheat hull flavonoids showed strong antioxidant activity in vivo and in vitro. Buckwheat hull flavonoids played a hepatoprotective role in vivo and in vitro.

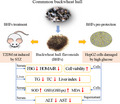

## 1 INTRODUCTION

Common buckwheat is one of the most commonly cultivated grain species across the globe. Large amounts of buckwheat hulls are produced during its processing as a by‐product. Numerous studies reveal that buckwheat hulls are embedded with rich amounts of flavonoids, which include but are not limited to, rutin, vitexin, quercetin, isoorientin, and hyperoside (Cui et al., 2020; Dziadek et al., 2016; Zhang et al., 2017). Flavonoids are the most studied subclass of polyphenolic compounds that are present in almost all parts of flowering plants, such as grains, fruits, vegetables, and teas (Cassidy & Minihane, 2017; Swallah et al., 2020), with more than 9,000 identified different structures bearing similar diphenol propane skeleton in nature (C6‐C3‐C6) (Swallah et al., 2020). Flavonoids are well evidenced in numerous studies as the widest subclass with hydrosoluble heterocyclic phenol substance (Swallah et al., 2021). Numerous studies demonstrated that flavonoids are beneficial to human's health due to their powerful pharmacological activities, including antidiabetic, antioxidant, anti‐inflammatory, anticancer, and antihypertensive. For example, recent studies have demonstrated that cherry flavonoids could inhibit lipid oxidation in mouse serum and tissues (Dong et al., 2021) and guava leaf flavonoids had significant antidiabetic and liver protective activities in diabetic mice (Zhu et al., 2020). Therefore, flavonoids are regarded as a unique class of therapeutic molecules and valued by the scientific community (Cassidy & Minihane, 2017; Maaliki et al., 2019; Zaragozá et al., 2020).

Public health data show that 463 million people worldwide are suffering from diabetes. It is predicted that the number of diabetics worldwide will reach 578 million (10.2% of the population) by 2030 (Williams & Colagiuri, 2019). Ninety percent of people with diabetes are suffering from type 2 diabetes mellitus (T2DM), characterized by high blood glucose level and insulin resistance. It can cause severe diabetic complications and cause further damage to organs and tissues. The liver is an essential organ of glucose metabolism in the body, involving glycolysis, gluconeogenesis, glycogen synthesis, and decomposition, which plays vital role in controlling normal glucose homeostasis (Zhao & Xing, 2017). The metabolic defects associated with diabetes often lead to changes in liver metabolism, which leads to liver toxicity and cell death. Although liver has good regeneration potential, long‐standing diabetes can manifest a spectrum of liver diseases, including altered liver functions, nonalcoholic fatty liver disease, end‐stage liver disease, and even hepatocellular carcinoma (Bedi et al., 2019). Meanwhile, diabetes also induces oxidative stress in the liver, which is characterized by an increase in the concentration of reactive oxygen species of tissue, resulting in a significant reduction of cellular antioxidants (Bedi et al., 2019; Lucchesi et al., 2013). On the other hand, although medical interventions achieve the goal of lowering blood sugar, one point that cannot be ignored is that they also aggravate burden on the liver. Therefore, it is necessary to discover a relatively safer component that lowers blood sugar in order to ease the liver's burden. There is no doubt that natural products rich in flavonoids have good potential to prevent diabetes. Progressively, more research endorses the potential application of buckwheat hull extracts in improving diabetes and antioxidant capacity (Dziadek et al., 2016; Park et al., 2019).

This paper aims to explore the hepatoprotective effects of buckwheat hull flavonoids (BHFs) in T2DM rats and HepG2 hepatocytes. Almost all reported studies on buckwheat hull flavonoids (BHFs) portray significant effect in reducing blood glucose, improving glucolipid metabolism (Li et al., 2019), and protecting diabetic nephropathy (Tang et al., 2019), where the influence of BHFs on liver cells and tissues is not well defined. Hence, this study will provide scientific results for buckwheat hull as a raw material and a functional food to prevent T2DM.

## 2 MATERIALS AND METHODS

### 2.1 Materials

Common Buckwheat (*Fagopyrum esculentum* Moench) hulls were purchased from Chifeng, Inner Mongolia, China.

### 2.2 Preparation of buckwheat hull flavonoids

**FIGURE 1:**
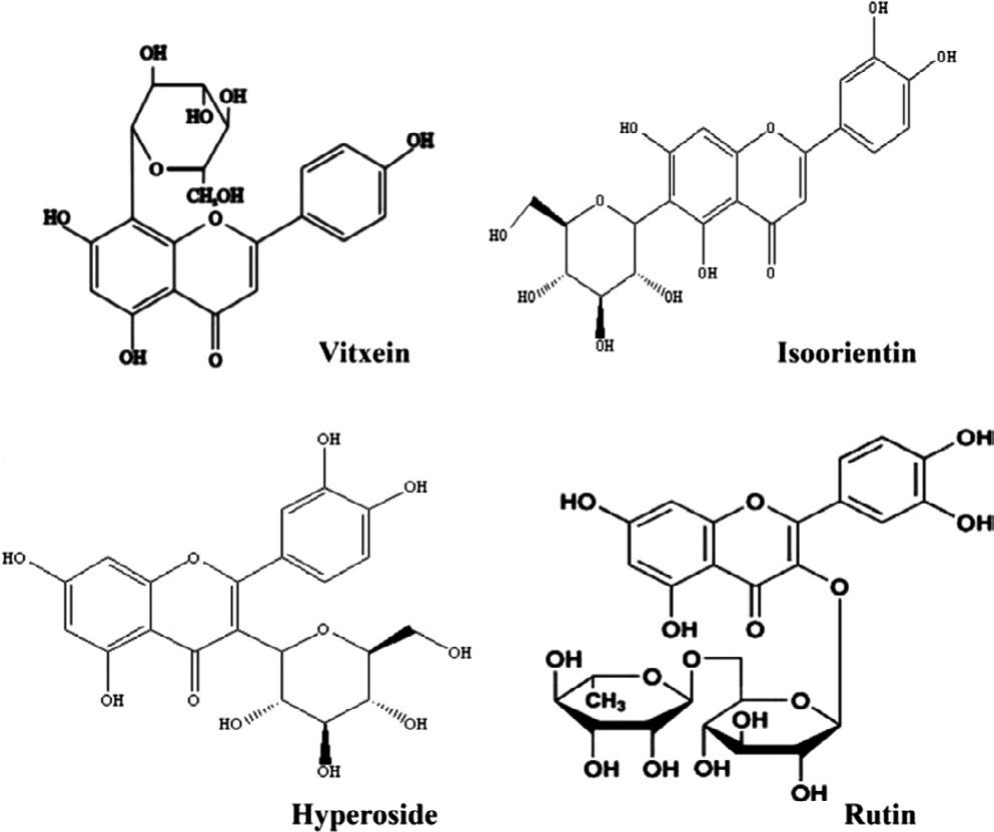
Four main components and chemical structure of buckwheat hull flavonoid

Buckwheat hull flavonoids (BHFs) was prepared from common buckwheat hull powder according to our previous studies (Li et al., 2019; Zhao et al., 2018). Briefly, the BHFs were extracted from buckwheat hull powder twice with deionized water (1:20, m/v) at 121°C for 20 min. The crude extract was adsorbed with macroporous resin D‐101 (Dong Hong chemical company), and BHFs were eluted by 70% ethanol after washing with distilled water. The purified crude product was then adsorbed with macroporous resin ADS‐7 (Dong Hong chemical company), and BHFs were eluted using 40% ethanol after washing with 10% ethanol. The total flavonoid content of BHFs is approximately 90% (determined using AlCl_3_‐NaNO_2_ colorimetry where rutin was used as a standard). Four main chemical components and structures of BHFs, including vitexin, isoorientin, hyperoside, and rutin, were identified (Figure 1) as reported in our previous study (Cui et al., 2020).

### 2.3 Evaluation of the hepatoprotective potential of BHFs in T2DM rats

#### 2.3.1 Animal model and experimental design

Five‐week‐old healthy male Wistar rats (120–150 g) were procured from Changchun Yisi Experimental Animal Technology Co., Ltd. The animals were housed under normal environmental conditions (temperature: 22 ± 3°C, humidity: 65 ± 5%, and 12 hr light/dark cycles) and were maintained with free access to water and a standard nutritionally balanced pellet diet (Changchun Yisi Experimental Animal Technology Co., Ltd). The T2DM rat model was established by multiple intraperitoneal injections of streptozotocin (STZ, 30 mg/kg.b.wt) after high‐sugar and high‐fat diets (Composition: 10% lard, 10% sucrose and 5% cholesterol) for 4 weeks with some modifications according to Zhang et al. (2008). Rats gradually showed polyphagia, polydipsia, and polyuria, with low spirits and yellow coat color. The glucose level was measured from the tail vein blood using glucose oxidase method with a blood glucometer (Johnson & Johnson (China) Investment Co., Ltd). Only rats with a fasting blood glucose (FBG) above 11.1 mmol/L were considered as T2DM, where they were randomly divided into three groups. The groups consisted of model group (distilled water, 10 ml/kg.b.wt/d, *n* = 8), BHFs group (BHFs, 50 mg/kg.b.wt/d, *n* = 8), and metformin group (metformin, 200 mg/kg.b.wt/d, *n* = 8). Meanwhile, normal rats were set as the control group (distilled water, 10 ml/kg.b.wt/d, *n* = 8). All rats were given a single oral gavage daily for 4 weeks.

#### 2.3.2 Samples collection and evaluation of liver index

During treatment, blood was sampled from the tail vein to monitor FBG on days 0, 7, 17, and 28. At the end of the experiment, rats were anesthetized by injection of 10% urethane. Blood samples were collected from the abdominal aorta and allowed to clot. The serum was separated for the determination of glucose levels and enzyme activities. The livers were excised and weighed after the animals were killed. Then, the liver samples were dissected immediately after rinsing with ice‐cold saline and stored at −80°C for evaluation of liver lipid parameters. The liver index, which was used to evaluate hepatomegaly, was expressed as follows: The liver index (mg/g) = (Liver weight (g)/Final body weight (g)) × 1,000.

#### 2.3.3 Determination of fasting blood glucose and fasting insulin levels

Serum glucose levels were measured using glucose oxidase method (Shanghai Hengyuan Biochemical Reagent Co., Ltd). Fasting insulin (FINS) levels were determined using an ELISA kit (Shanghai Hengyuan Biochemical Reagent Co., Ltd) following the manufacturer's instructions. A homeostasis model assessment of insulin resistance (HOMA‐IR) (Bowe et al., 2014), a method to quantify insulin resistance, was used following the formula: HOMA‐IR = FBG (mU/L) × FINS (mmol/L)/22.5.

#### 2.3.4 Oral glucose tolerance test

On the 26th day of BHFs administration, oral glucose tolerance test (OGTT) was performed in all rats after fasting (with water supply) for 12 hr. The rats were gavaged with 10% glucose (2.5 g/kg.b.wt), and the blood samples were collected from the tail vein at 0, 30, 60, and 120 min for the determination of blood glucose level. Glucose tolerance was quantified as the area under the curve (AUC) integrated from 0 to 120 min using the trapezoidal method (Bowe et al., 2014).

#### 2.3.5 Determination of serum oxidative stress markers levels

Superoxide dismutase (SOD) and catalase (CAT) activities and glutathione (GSH) and malondialdehyde (MDA) levels in the serum were measured using commercially available kits (Nanjing Jiancheng Bioengineering Institute) according to manufacturer's instructions.

#### 2.3.6 Determination of hepatic lipid levels

The hepatic triglyceride (TG) and total cholesterol (TC) levels of the liver were measured using commercially available kits (Nanjing Jiancheng Bioengineering Institute) according to manufacturer's instructions.

#### 2.3.7 Determination of serum liver function markers

Serum hepatocyte growth factor (HGF), alanine aminotransferase (ALT), and aspartate aminotransferase (AST) levels were measured using commercially available kits (Nanjing Jiancheng Bioengineering Institute) according to manufacturer's instructions.

### 2.4 Hepatoprotective potential of BHFs against high glucose‐damaged HepG2 cells

#### 2.4.1 Cytotoxicity and cytoprotective potential of BHFs

Human hepatoblastoma (HepG2) cell line was kindly provided from the College of Life Sciences, Jilin Agricultural University, Changchun, China. The cells were incubated in high glucose (4.5 g/L of glucose) Dulbecco's modified Eagle's medium (DMEM, Gibco) containing 10% fetal bovine serum (FBS, Biological Industries Co. Ltd.), 100 U/ml penicillin, and 100 µg/ml streptomycin at 37°C in a humidified atmosphere of 5% CO_2_. For cytotoxicity assessment, HepG2 cells were seeded in 96‐well plates at the density of 2 × 10^4^ cells/well and cultured with a maintenance medium for 24 hr. Then, they were exposed to high glucose (100, 200, 400, and 800 mM) for 12, 24, and 48 hr or treated with BHFs at the concentrations of 1, 10, 25, 50, and 100 µg/ml for 24 hr. To preliminarily assess the cytoprotective potential, HepG2 cells were preincubated with biologically safe concentrations of BHFs (10, 25, and 50 µg/ml) for 24 hr and exposed to high glucose concentration (200 mM) for 24 hr. Cell viability was measured by MTS assay according to manufacturer's instructions, and the absorbance was measured at 512 nm using a microplate reader (Tecan Infinite M200, Tecan).

#### 2.4.2 Morphological observation and determination of oxidative stress markers and transaminase levels

HepG2 cells were seeded in 6‐well plates at the density of 3 × 10^5^ cells/well. After 24 hr, cells were treated as described above. Morphological changes were observed using an inverted microscope (Leica DM IL LED) at 400× magnification. Supernatant medium was collected to determine leakage of ALT and AST using commercial kits (Nanjing Jiancheng Bioengineering Research Institute) according to manufacturer's instructions. Meanwhile, cells were washed extensively with ice‐cold PBS, where 300 µl 1% Triton X‐100 (Solarbio) was added to each well, lysing for 30–40 min. SOD, glutathione peroxidase (GSH‐Px), and CAT activities and MDA levels were measured using appropriate assay kits (Nanjing Jiancheng Bioengineering Research Institute) according to manufacturer's instructions.

### 2.5 Statistical analysis

GraphPad Prism version 7.0 (GraphPad Software) was used in all charts and analyses. Comparison of differences among different groups was performed using one‐way analysis of variance (ANOVA) followed by Dunnett's test as a post hoc analysis. All data were presented as mean ± standard deviations (*SD*) of more than three independent experiments. Statistical significance levels are presented as *p < .05, **p < .01, ***p < .001, and ****p < .0001. *p* values < .05 were considered to be statistically significant.

## 3 RESULTS

### 3.1 Effects of BHFs on glucose and insulin levels in T2DM rats

**FIGURE 2:**
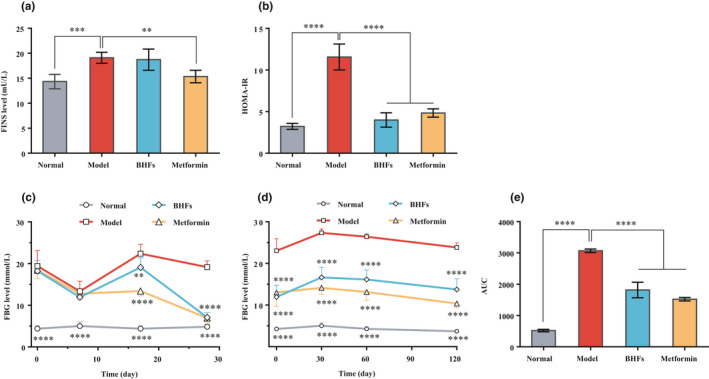
Effects of BHFs on glucose and insulin levels in serum of diabetic rats induced by STZ. (a) Fasting Insulin (FINS), (b) HOMA‐Insulin Resistance (HOMA‐IR), (c) Fasting blood glucose (FBG), (d) Oral glucose tolerance test (OGTT), and (e) Area under the curve (AUC). All data were presented as mean ± *SD*, *n* = 8, as compared to model, **p < .01; ***p < .001, ****p < .0001

To determine the ability of BHFs to control blood glucose level in T2DM rats, FINS, FBG, and OGTT were monitored. As shown in Figure 2, compared with normal rats, the levels of FINS and FBG, as well as HOMA‐IR, in diabetic model rats increased significantly (*p* < .001). While the high insulin levels did not show significant improvement in diabetic rats under the therapeutic intervention (Figure 2a), the interventions of BHFs and metformin significantly reduced HOMA‐IR by 65.50% and 58.20%, respectively, compared with the diabetic rats (Figure 2b, *p* < .0001). Furthermore, after drug intervention for 4 weeks, the FBG of BHFs group decreased from 18.30 on day 0 to 8.40 mmol/L on day 28, almost approaching normal levels, whereas untreated rats exhibited a steady trend (Figure 2c). Oral glucose tolerance test (OGTT) was used to simplify and facilitate the diagnosis of diabetes (Rahman et al., 2018). Results showed that the glucose tolerance ability of the model group was significantly lower than the normal group. In comparison, a better glucose tolerance ability was observed in BHFs and metformin groups, which showed a significant improvement of glucose tolerance at 120 min of AUC by 40.79% and 50.41%, respectively (Figure 2d,e, *p* < .0001). These findings confirmed that BHFs have an anti‐hyperglycemic effect comparable to metformin in STZ‐induced T2DM rats.

### 3.2 Effects of BHFs on serum antioxidant markers in T2DM rats

**FIGURE 3:**
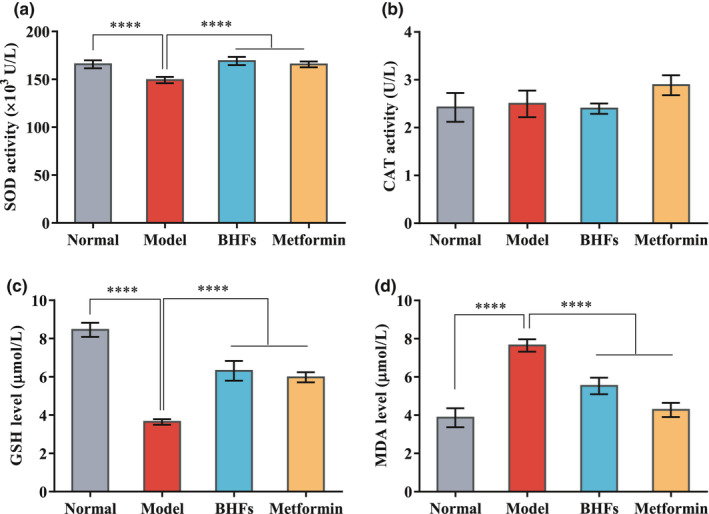
Effects of BHFs on antioxidant markers levels in serum of diabetic rats induced by STZ. (a) Superoxide dismutase (SOD) activity, (b) catalase (CAT) activity, (c) reduced glutathione (GSH) level, and (d) malondialdehyde (MDA) level. All data were presented as mean ± *SD*, *n* = 8, as compared to the model, ****p < .0001

Accumulating evidence indicated that oxidative stress induced by chronic hyperglycemia is considered a major factor contributing to the development of diabetic pathological process with liver damage (Kayama et al., 2015; Keane et al., 2015; Mohamed et al., 2016). To investigate whether BHFs acts as an antioxidant in the treatment of diabetes, the SOD and CAT activities and GSH and MDA levels were measured. In Figure 3, lower serum SOD activity and GSH level were observed in diabetic model rats (*p* < .0001). However, BHF treatment significantly improved SOD activity (Figure 3a) and GSH level (Figure 3c) (*p* < .0001). CAT activity remained unchanged after all treatments (Figure 3b). MDA level, a biomarker of oxidative damage and lipid peroxidation in the model rats, was markedly elevated compared with the normal group (Figure 3b, *p* < .0001). BHFs and metformin treatment reduced MDA levels dramatically by 27.73% and 44.11%, respectively, compared with the model group (Figure 3d). These results indicated that BHFs showed strong antioxidant activity in T2DM rats.

### 3.3 Effects of BHFs on liver index, lipid profiles, and serum hepatic injury markers in T2DM rats

**FIGURE 4:**
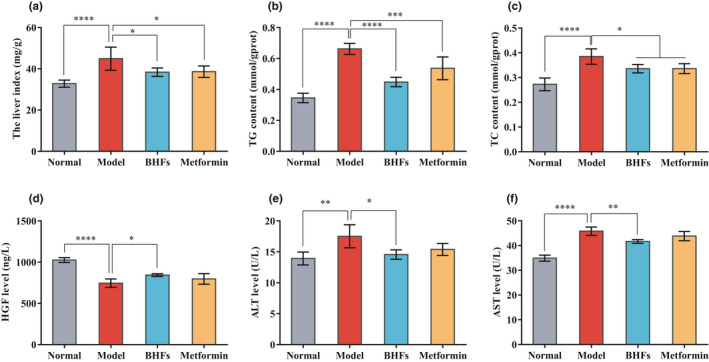
Effects of BHFs on the liver index, liver lipid, and serum hepatic injury markers levels in diabetic rats induced by STZ. (a) The liver index, (b, c) triglyceride (TG) and total cholesterol (TC) content in liver, (d, e, f) hepatocyte growth factor (HGF), alanine aminotransferase (ALT), and aspartate aminotransferase (AST) levels in serum. All data were presented as mean ± *SD*, *n* = 8, as compared to model, *p < .05, **p < .01; ***p < .001, ****p < .0001

Liver damage is one of the main complications of T2DM. Hepatomegaly characterizes the pathological consequence of liver damage and fat infiltration caused by heterotopic accumulation of lipids with elevation of various liver enzymes. Compared with normal rats, liver index, TG and TC levels, and ALT and AST activities increased significantly in STZ‐induced diabetic rats, while serum HGF level decreased significantly (Figure 4, *p* < .01). BHF treatment significantly reduced the liver index by 14.60% and decreased TG and TC levels of liver tissues by 32.35% and 12.75%, respectively, compared with the model group (Figure 4a–c, *p* < .05). A similar pattern was also observed in the case of metformin administration, and it significantly decreased the liver index by 14.04%, the TG and TC by 18.93% and 12.73%, respectively (Figure 4a–c, *p* < .05). It is worth noting that, compared with the model group, BHFs administration significantly reduced serum ALT and AST levels by 16.84% and 9.04%, respectively, and increased the HGF levels by 13.18% (*p* < .05), while metformin treatment had no effect on liver enzymes and HGF level (*p* > .05) (Figure 4d–f). Thus, BHFs played a positive role in hepatoprotection.

### 3.4 Effects of BHFs or high glucose on cytotoxicity and cytoprotection in HepG2 cells

**FIGURE 5:**
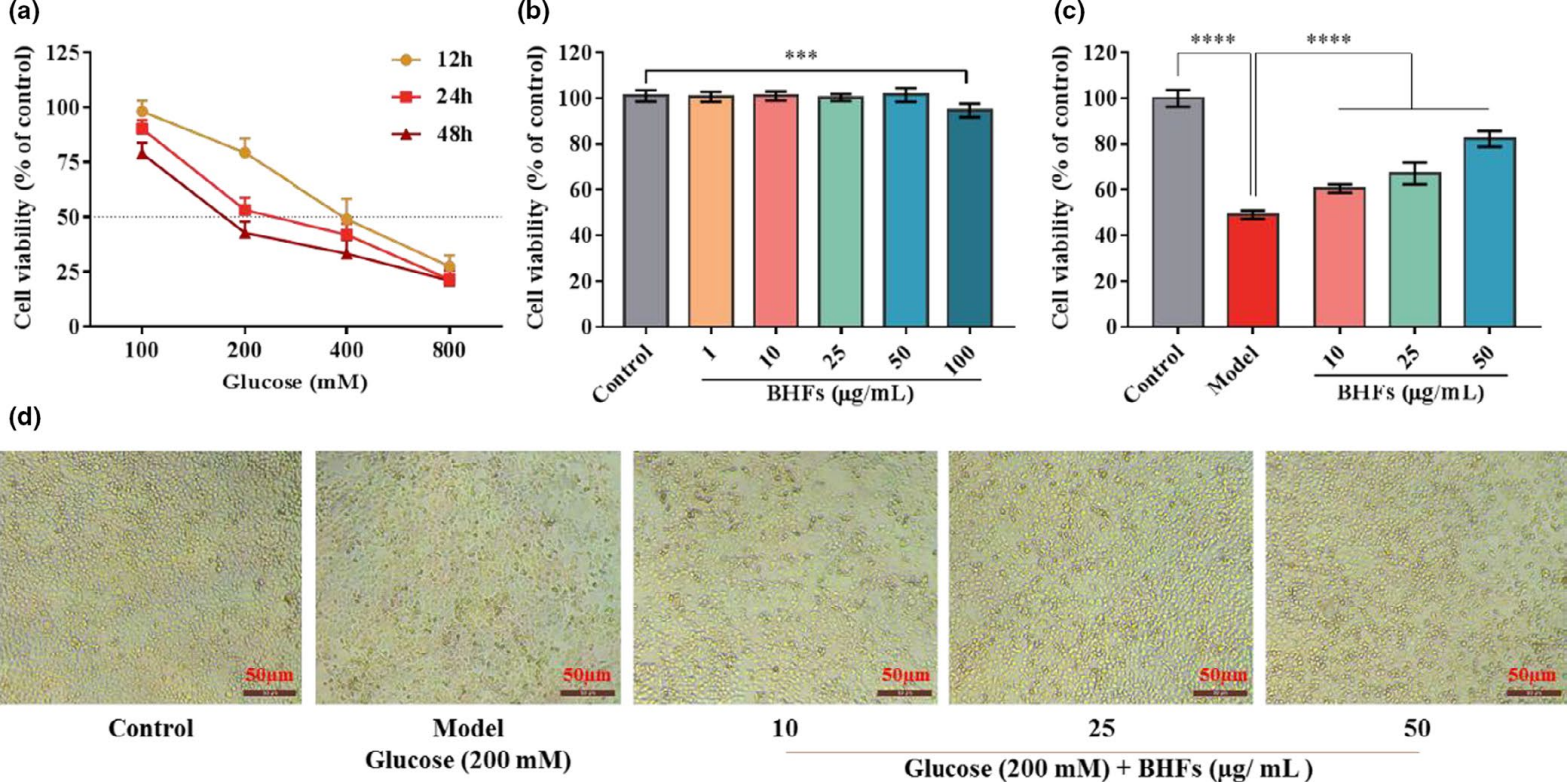
Effects of BHFs or high glucose on cytotoxicity and cytoprotection in HepG2 cells. (a) The cytotoxicity of HepG2 cells following the exposure of various concentrations of high glucose for 12, 24, and 48 hr. (b) The cytotoxicity of HepG2 cells incubated with various concentrations BHFs for 24 hr. (c) The cell viability of HepG2 cells following BHFs protection and high glucose damage. (d) The morphological changes of HepG2 cells following BHFs protection and high glucose damage. Original magnification, × 400. All data were presented as mean ± *SD*, *n* = 6 at least, as compared to model, ***p < .001 and ****p < .0001

The cytotoxic effects and cytoprotective potential of BHFs (or high glucose) have been assessed by MTT assay and observing morphological changes. Results showed that high glucose‐induced cell death at a dose‐ and time‐dependent manner. Cells treated with 200 mM glucose for 24 hr showed moderate injuries, suggesting that this condition was suitable to establish a high glucose damage model and was used for subsequent experiments (Figure 5a). Meanwhile, BHFs did not cause any effects on the cell viability of HepG2 cells at 10–50 µg/ml concentrations (Figure 5b, *p* > .05). In assessing the protection against high glucose damage, a dose‐dependent manner enhancement of cell viability was observed in HepG2 cells pretreated with BHFs at 10, 25, and 50 µg/ml for 24 hr (Figure 5c, *p* < .0001). Meanwhile, the characteristic morphological changes demonstrated that high glucose caused cell shrinkage and loss of adhesion capacity compared with the control. However, pre‐protection of BHFs restored their original morphology similar to the control at a concentration‐dependent manner. These results suggested the preventive potential of BHFs against high glucose‐induced damage (Figure 5d).

### 3.5 Effects of BHFs on antioxidant and liver function markers in HepG2 cells

**FIGURE 6:**
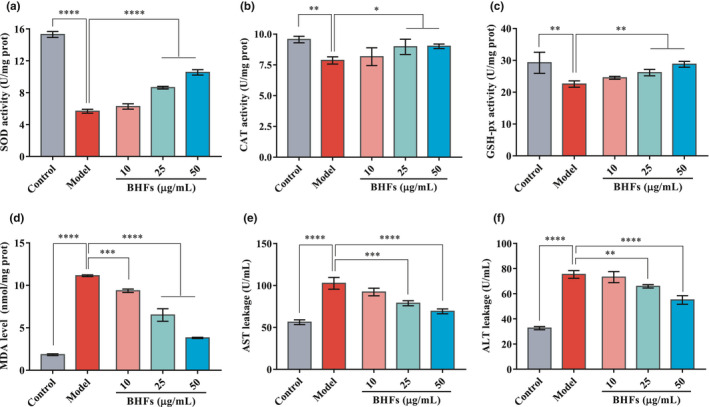
Effects of BHFs on antioxidant and liver function markers levels in HepG2 cells. (a) Superoxide dismutase (SOD) activity, (b) catalase (CAT) activity (c) glutathione peroxidase (GSH‐px) activity, (d) malondialdehyde (MDA) level, (e, f) aspartate aminotransferase (AST), and alanine aminotransferase (ALT) leakage in supernatant medium. All data were presented as mean ± *SD*, *n* = 3, as compared to model, *p < .05, **p < .01, ***p < .001, and ****p < .0001

To evaluate the pre‐protection effects of BHFs on antioxidant defense systems under high glucose pressure, the SOD, GSH‐Px, CAT activity, and MDA level, as well as the AST and ALT leakage, were measured in HepG2 cells exposed to high glucose level. As shown in Figure 6, after the unprotected HepG2 cells of the model group were exposed to high glucose (200 mM) for 24 hr, the antioxidant enzyme activity was severely damaged. At the same time, the oxidation product MDA and the leakage of AST and ALT increased significantly as compared to the control (*p* < .01). However, the BHFs pretreatment protected the HepG2 cells from high glucose‐induced damage at a dose‐dependent manner, where SOD, GSH‐Px, and CAT activities were significantly higher at 25 and 50 µg/ml concentrations than the unprotected group (model), accompanied by a significant reduction of MDA level (Figure 6a–d, *p* < .01). In terms of liver function, BHFs pretreatment reduced the release of AST and ALT in the cells at a dose‐dependent manner (*p* < .01). The AST and ALT leakages of the high‐dose pretreatment group (50 µg/ml) were reduced by 32.54% and 26.94%, respectively, compared with the model group (Figure 6e,f). These results demonstrated that BHFs have antioxidant and hepatoprotective potential against high glucose‐damaged HepG2 cells.

## 4 DISCUSSIONS

Liver disease, including nonalcoholic fatty liver disease (NAFLD), abnormal liver enzymes (ALT and AST), cirrhosis, and hepatocellular carcinoma, is an important cause of death in T2DM. Oxidative stress is part of the mechanism implicated in the pathogenesis of chronic diabetic complications as well as in liver dysfunction (Bedi et al., 2019; Lucchesi et al., 2013). T2DM can cause liver lipid deposition, where excessive lipid deposition exacerbates lipid oxidation, resulting in oxidative stress and inflammatory factor infiltration, enhancing the development of insulin resistance as well as impairing liver function (Pierantonelli & Svegliati‐Baroni, 2019; Sun et al., 2020). This study has proven that BHFs protected the liver effectively, both in T2DM rats and HepG2 cells.

Increasing studies indicated that improved glycemic control reduced the risk of diabetic complications, such as microvascular complications (Alkhalidy et al., 2018). This significant glucose controlling effect of flavonoids might be closely related to their multiple effects, such as anti‐amylase activity (Zhao et al., 2018), increasing proliferation of pancreatic β‐cells, and promoting insulin secretion (Hajiaghaalipour et al., 2015). Many in vitro and in vivo studies have shown beneficial effects of flavonoids on glucose homeostasis (Hajiaghaalipour et al., 2015). To investigate the mechanisms underlying the protection against liver dysfunction by the intervention of BHFs, serum FINS and FBG levels and glucose tolerance of T2DM rats were determined. Results demonstrated that BHFs were as effective as metformin in controlling blood glucose in T2DM rats (Figure 2). This result was highly consistent with previous studies in *db/db* mice (Li et al., 2019), suggesting BHFs can be a significant therapeutic component for lowing blood glucose in T2DM.

The liver is the main organ for maintaining glucose homeostasis (Nishida, 2017). As a collection of insulin‐sensitive tissues, liver is one of the main organs susceptible to the effects of chronic high glucose‐induced oxidative stress, which may lead to liver tissue injury with a vicious circle (Manna et al., 2010; Palsamy et al., 2010). One concern is that T2DM is often accompanied by abnormal fat levels and lipoprotein metabolism disorders. Dyslipidemia is directly or indirectly involved in the pathogenesis of liver disease by inducing insulin resistance and hepatic steatosis (Bedi et al., 2019). After 28 days of treatment, T2DM rats treated with BHFs showed a significant decrease in TG and TC levels compared with the untreated diabetic rats.

Moreover, BHFs significantly ameliorated ALT and AST levels (Figure 4), which are common in T2DM patients. These positive effects were above metformin, the most widely used first‐line therapy for T2DM (Zheng et al., 2015), suggesting the superiority of BHFs. The hepatoprotective effects of BHFs were also investigated in HepG2 cells against high glucose damage, where a dose‐dependent manner enhancement in cell viability was observed (Figure 5). The pre‐protection of BHFs significantly reduced AST and ALT leakage in the supernatant medium (Figure 6). These results demonstrated that BHFs has hepatoprotective potential against high glucose damage. On the other hand, growing evidence suggests that NAFLD and chronic kidney disease (CKD) share common pathogenic mechanisms and potential therapeutic targets (Musso et al., 2016). We had also studied the improvement of BHFs in diabetic nephropathy (DN) and found a significant reduction of inflammation and detected a great number of the downregulated differentially expressed proteins (DE protein) associated with the immune system, transport, and catabolism as well as environmental information processing (Tang et al., 2019). Such data would provide a prospective way to explore the mechanism of BHFs in NAFLD.

Increased oxidative stress is the main risk factor leading to insulin resistance and impaired glucose tolerance, ultimately leading to T2DM (Tangvarasittichai, 2015). Chronic high glucose conditions can induce oxidative stress by several mechanisms, such as, glucose autoxidation, polyol pathway, AGE formation, and PKCβ1/2 kinase, worsening glucose intolerance and impairing insulin signaling (Tangvarasittichai, 2015). The oxidatively modified by‐products of macromolecules are viewed as oxidative stress biomarkers in vivo and in vitro. Recent studies showed that once NAFLD is established in T2DM, the already compromised liver could be affected by ROS, and hepatocellular damage may increase with inflammation, progressing to steatohepatitis and cirrhosis (Lucchesi et al., 2013). Numerous research indicates that flavonoids derived from plants are beneficial for the liver. For instance, flavonoids from Wushan Shencha, which comes from the Three Gorges Valley of Middle Yangtze River in China, can regulate liver function indexes and inflammatory cytokines levels, regulating oxidative stress in liver tissues (Zhu et al., 2019). Similar results were observed in our study. We found that BHF treatment notably increased the SOD activity and GSH levels in the serum, whereas the MDA levels were reduced significantly compared with that of the untreated diabetic rats (Figure 3). Pretreatment of BHFs protected the HepG2 cells from high glucose‐induced damage at a dose‐dependent manner with the increasing levels of SOD, GSH‐Px, and CAT activities accompanied by a significant reduction of MDA level.

Flavonoids are well known for their antioxidant properties (Alkhalidy et al., 2018; Cassidy & Minihane, 2017). We found that flavonoids from buckwheat hull exert high antioxidant capacity, due to the presence of BHFs or flavonoid monomers derived from buckwheat hull (Cui et al., 2020). In that study, we observed that total flavonoids and monomeric flavonoids showed similar antioxidant activity. In some indicators, the monomer activity was even higher in vitro. However, at the cellular level, it was found that BHFs had better antioxidant effect than the monomeric flavonoids in H_2_O_2_‐induced HepG2 cells indicating the synergistic interaction of flavonoids. In this experiment, we strongly confirmed that BHFs significantly improved liver oxidative stress indicators in the liver of T2DM rats and HepG2 cells, which was another important evidence that BHFs have expectant antioxidative stress capabilities.

## 5 CONCLUSION

This study demonstrated positive results that buckwheat hull flavonoids improved insulin resistance, blood glucose level, and oxidative stress state and showed a significant protective potential against liver fatty damage in T2DM rats. Meanwhile, the pre‐protection of BHFs can maintain HepG2 cells at higher cell viability and protected cells from oxidative damage caused by chronic high glucose. In summary, our findings suggest that BHFs may be a safe and effective food adjuvant for the treatment of T2DM and its complications, such as liver injury.

## CONFLICT OF INTEREST

The authors declare that they do not have any conflict of interest, and the manuscript is approved by all authors for publication.

## AUTHOR CONTRIBUTION

**Hai Wang:** Conceptualization (equal); Formal analysis (equal); Investigation (equal); Methodology (equal); Writing‐original draft (lead); Writing‐review & editing (equal). **Shuyan Liu:** Formal analysis (equal); Investigation (equal); Methodology (equal); Software (equal); Validation (lead); Visualization (equal). **Yang Cui:** Methodology (equal); Supervision (equal); Validation (equal). **Yue Wang:** Formal analysis (supporting); Investigation (equal); Methodology (equal); Software (supporting); Validation (equal). **Yang Guo:** Investigation (supporting); Methodology (supporting); Validation (equal). **Xiujuan Wang:** Supervision (equal); Writing‐review & editing (equal). **Junmei Liu:** Conceptualization (equal); Formal analysis (lead); Funding acquisition (equal); Supervision (equal); Writing‐review & editing (equal). **Chunhong Piao:** Conceptualization (lead); Data curation (equal); Funding acquisition (lead); Methodology (equal); Project administration (equal); Resources (equal); Supervision (lead); Writing‐original draft (equal); Writing‐review & editing (lead).

## ETHICAL REVIEW

This study's protocols and procedures were ethically reviewed and approved by the Animal Ethics Committee of Wish Technology with the ethical code: IACUC‐002‐F‐2019006. This study does not involve any human or patients testing.

## INFORMED CONSENT

Written informed consent was obtained from all study participants.

## Data Availability

The data that support the findings of this study are available from the corresponding author upon reasonable request.
